# Smart Multi-Responsive Biomaterials and Their Applications for 4D Bioprinting

**DOI:** 10.3390/biomimetics9080484

**Published:** 2024-08-11

**Authors:** Jinku Kim, Gouripriya D A, Poonam Debnath, Prosenjit Saha

**Affiliations:** 1 Department of Biological and Chemical Engineering, Hongik University, Sejong 30016, Republic of Korea; 2 Center for Interdisciplinary Science (CIS), JIS Institute of Advanced Studies and Research (JISIASR), JIS University, Kolkata 700091, India; gourip2020@gmail.com (G.D.A.); poonamdebnath1912@gmail.com (P.D.)

**Keywords:** 4D printing, biomaterials, smart structures, tissue engineering, regenerative medicine

## Abstract

The emergence of 4D printing has become a pivotal tool to produce complex structures in biomedical applications such as tissue engineering and regenerative medicine. This chapter provides a concise overview of the current state of the field and its immense potential to better understand the involved technologies to build sophisticated 4D-printed structures. These structures have the capability to sense and respond to a diverse range of stimuli, which include changes in temperature, humidity, or electricity/magnetics. First, we describe 4D printing technologies, which include extrusion-based inkjet printing, and light-based and droplet-based methods including selective laser sintering (SLS). Several types of biomaterials for 4D printing, which can undergo structural changes in various external stimuli over time were also presented. These structures hold the promise of revolutionizing fields that require adaptable and intelligent materials. Moreover, biomedical applications of 4D-printed smart structures were highlighted, spanning a wide spectrum of intended applications from drug delivery to regenerative medicine. Finally, we address a number of challenges associated with current technologies, touching upon ethical and regulatory aspects of the technologies, along with the need for standardized protocols in both in vitro as well as in vivo testing of 4D-printed structures, which are crucial steps toward eventual clinical realization.

## 1. Introduction

Three-dimensional (3D) bioprinting is an advanced technology to produce complex customized 3D shapes with micro porous structure built by the deposition of biomaterials with a layer-by-layer manner to mimic human tissues or organs [1]. The 3D bioprinting method has become a critical tool to fabricate a complex 3D extracellular matrix (ECM) for various biomedical applications such as tissue engineering and regenerative medicine (TERM) [2]. A notable development in this field has been the evolution of 3D to 4D bioprinting, which is an advanced technique allowing a 3D printed structures to respond to various external stimuli (e.g., temperature, humidity, or electricity/magnetics) over time. Therefore, by introducing the additional dimension of time into the 3D printing process, 4D bioprinting has been able to revolutionize the fields for the creation of smart multi-responsive structures with transformative potential across various biomedical applications [3,4].

This review describes a focused overview of smart biomaterials and their biomedical applications for 4D bioprinting. We delve into the fundamental principles that underlie 4D bioprinting, examining how it builds upon the foundations of 3D printing and extends them into a dynamic realm. Through a detailed analysis of materials and biomedical applications that are an essential component for 4D bioprinting, we aim to provide a holistic understanding of the field’s current state and its enormous potential. The multi-responsiveness inherent in 4D-printed structures is a hallmark of their ingenuity. These structures have the capability to sense and respond to a variety of stimuli including temperature, humidity, electricity/magnetics, light, pH, or the presence of specific chemicals [5]. This responsiveness is a reflection of the careful integration of advanced materials and precise fabrication techniques, resulting in smart structures that can adapt and transform in ways that have previously been reserved for the natural world. Moreover, biomedical applications of 4D-printed smart structures span a wide spectrum of biomedical applications, such as health-care applications and regenerative medicine. These structures hold the promise of revolutionizing fields that require adaptable and intelligent materials [6,7]. Their potential to create self-assembling medical implants, dynamically changing architectural elements, and shape-shifting devices will place 4D bioprinting as a game-changer in the pursuit of organ transplants.

As we begin this journey through the world of 4D-printed smart, multi-responsive structures, we aim to provide a better understanding of the intricacies of this rapidly evolving field. Herein, we will excavate a deeper understanding into technologies, materials, and the plethora of applications that showcase the remarkable potential of 4D bioprinting. By the end of this exploration, we hope that readers will gain a profound appreciation for the transformative power of engineering structures that come alive in the fourth dimension.

Embarking on the journey of 3D bioprinting and transitioning to 4D bioprinting presents a significant challenge: the need to optimize multiple variables. It involves the optimization of materials, printing parameters, and the selection of cells. Moreover, it is important to consider the relevance of the technology to clinical applications, including the needs of clinicians and patients. Achieving this requires translational research, which can be difficult in a new field where regulatory processes are also not well-established.

## 2. Types of 4D Printing Technologies

The concept of 4D bioprinting draws inspiration from nature’s own dynamic processes. As living organisms respond and adapt to their environments, 4D-printed structures can autonomously undergo shape changes, self-assembly, or functional alterations in response to external stimuli such as humidity, temperature, electricity and so on. This ability to harness the fourth dimension, time, enables a new era of possibilities for engineering complex structures that mirror the intricate behaviors of natural systems [3]. However, the current 4D printing technologies are generally acknowledged as 3D bioprinting. The 4D bioprinting combining 3D bioprinting with stimuli responsive biomaterials, has emerged to develop structures that can dynamically transform their shape or function in response to physical, chemical, and biological stimuli. Thus, 4D printing offers significant potential for constructing complex structures that can dynamically interact with their environment, enhancing their functionality and adaptability [3]. One of the easiest mechanisms for 4D bioprinting involves the combination of heterogeneous materials that possess unique properties and responsiveness to different stimuli [4]. For example, Naficy and co-workers fabricated a complex 3D structure with two different materials: one was responsive to moisture, which was poly (2-hydroxyethyl methacrylate) (HEMA) and the other responsive to temperature, which was poly (*N*-propylacrylamide) (NIPAM). As a result, they were able to create complex structures using these hydrogels by inkjet printing. By hydration and a change in temperature, the structure revealed the reversible shape transformation [5].

There are several types of available 4D-printing technologies, each of which has its own advantages and limitations (Table 1). The most widely used printing methods are extrusion-based methods, also known as fused filament fabrication (FFF) or fused deposition modeling (FDM). This technique is used to create complex 3D objects by precisely depositing bioinks in a layer-by-layer manner with computer-controlled movements. The extrusion-based methods are widely available for 4D printing, relatively easy to use, and can print a wide range of bioinks, including cell-laden hydrogels [6]. Due to the layer-by-layer nature of this technology, the resolution is limited by the nozzle diameter, typically in the range of 100–200 μm, thus, achieving high resolution of resulting structures can be difficult due to the viscosity of bioinks and the extruding mechanism [7]. Another limitation of the extrusion-based method is the structural integrity since maintaining the shape and structural stability of printed structures can be challenging, particularly with softer materials [8]. In addition, it is difficult to print multiple materials using this method because different thermoplastics may have different printing temperatures. Overall, extrusion-based 3D bioprinting is a powerful and versatile technology in the field of biofabrication, enabling the creation of functional biological structures for a variety of biomedical applications.

Inkjet printing, on the contrary, can be used for multi-material printing if two polymers are compatible with each other [13]. Inkjet 3D printing operates on principles similar to traditional 2D inkjet printing and uses multiple printheads to simultaneously deposit different photocurable droplets of bioinks to form a complex 3D structure, followed by photocuring. This enables the convenient 3D fabrication composed of multi-materials at a relatively high resolution, approximately 30~40 and 200~400 μm for single-material and multi-material printing, respectively [14]. This method also has high material efficiency with minimal waste due to precise droplet placement [9]. However, bioinks of inkjet 3D-printing processes must have low viscosity to be printable, which can limit the range of usable materials and the printers can be expensive, particularly those capable of multi-material printing [15]. This technology is also limited by nozzle clogging, requiring careful formulation of bioinks and maintenance of the printhead [10].

Another fast-growing method for 3D printing technologies is the so-called vat photopolymerization (or light-based printing). This technology can be called stereolithography (SLA) or digital light processing (DLP), depending on the light sources (laser beam or digital light projector is used for photocuring). Stereolithography, a 3D printing technique, which employs photopolymerization to build successive polymeric layers to form a 3D polymeric network. Stereolithography (SLA) 3D printing is a highly precise and versatile technology widely used for producing detailed and accurate 3D structures. Its ability to create smooth surfaces and complex geometries makes it valuable in various industries, including prototyping, medical modeling, dental applications, and jewelry design. Despite its challenges, such as material limitations and post-processing requirements, SLA has become an increasingly popular choice for applications requiring high resolution and fine detail [11].

The last major group of polymer 3D printing methods is the droplet–based (or jetting-based) methods including selective laser sintering (SLS), which is a versatile 3D printing technology that creates 3D objects by selectively fusing powdered material using a high-power laser [13]. Selective Laser Sintering (SLS) is a robust and versatile 3D printing technology well-suited for producing durable, complex parts with good mechanical properties. Its ability to process a wide range of materials and produce parts without the need for support structures makes it ideal for both prototyping and end-use applications across various industries. Despite challenges such as surface finish and powder handling, SLS also remains a popular choice for high-quality, functional 3D printing [6].

## 3. Biomaterials for 4D Bioprinting

4D bioprinting uses smart materials such as shape memory materials capable of responding to external stimuli including light, voltage, temperature, magnetic, pH or other stimuli [14]. Stimuli-responsive smart materials have drawn a lot of attraction in obtaining the desired architecture and functionality of 4D-printed structures. Biomaterials can be classified as natural or synthetic materials and they are intended to interact with living tissues, thereby should not be toxic or not cause any harm to the recipient [16]. Various types of smart biomaterials have been explored as suitable candidates for 4D printing applications and they exhibit distinct properties and responsiveness to stimuli [17]. Table 2 summarizes biomaterials responding to respective external stimuli and their behaviors as well as applications.

### 3.1. Humidity-Responsive Biomaterials

**Figure 1:**
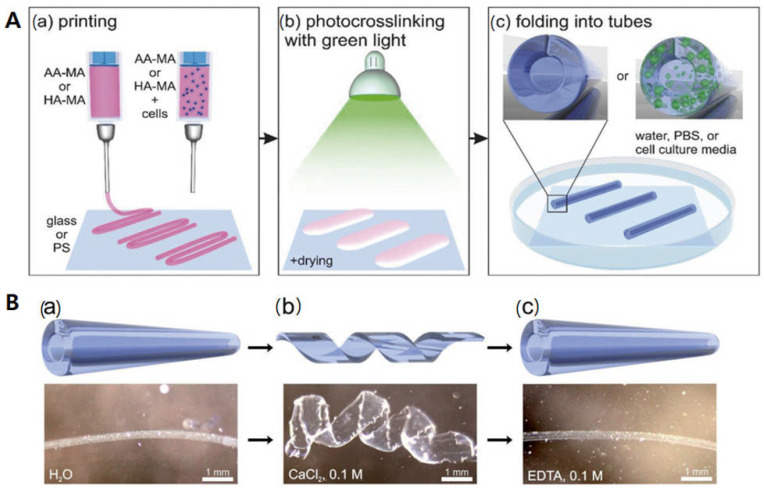
(**A**) (**a**) Four-dimensional bioprinting of cell-laden structures fabricated by moisture-responsive methacrylated alginate (AA-MA) or hyaluronic acid (HA-MA) hydrogels. (**b**) Green light was used for mild drying of structures. (**c**) Instant folding into tubes obtained upon immersion of crosslinked films in water, PBS or cell culture media. (**B**) The tube responsiveness; upper panel (cartoon), lower panel (representative images in water). (**a**) The same tube immersed in CaCl_2_ solution, (**b**) additional crosslinking of alginate with Ca^2+^ ions led to unfolding of the tube, and (**c**) folded tube immersed in EDTA solution. Reproduced with permission [36].

Water is one of the most common stimuli for printed structures since many biological systems can change their shapes in response to variations in the surrounding humidity. Humidity-responsive materials can transform their shape and size accordingly when exposed to water (moisture). However, it is crucial to precisely control the degree of transformation of the materials during the transition to maintain the integrity of printed constructs [3]. Such biomaterials have already found their usefulness in 4D bioprinting. For example, Ionov and colleagues developed bioink hydrogels comprised of alginate and hyaluronic acid [36]. First, self-folding hydrogel-based tubes with/without live cells were printed onto different substrates (Figure 1(Aa)). Following the printing, the films were photopolymerized with green light to generate a composite hydrogel (Figure 1(Ab)). After immersion of the crosslinked films in water, they instantly folded into tubes (Figure 1(Ac)). In the case of cell laden tubes, the cells were viable and evenly distributed throughout the self-folding tubes. The authors demonstrated that self-folding of the printed film can be formed into tubes with the crosslinking gradient in the films since the top layer of the film absorbs more light than the bottom, leading to different volume expansions. In addition, the printed tubes can demonstrate a reversible folding and unfolding behavior with/without Ca^2+^ ions (Figure 1B). They can swell in the presence of Ca^2+^ ions, but do not swell in the absence of Ca^2+^ ions. Similarly, Thiele and colleagues developed a moisture-responsive cellulose-based material [37]. They produced self-standing films using cellulose stearoyl esters (CSEs) with a varying degree of substitution (DS) on each side. The films with low DS (CSE0.3) revealed moisture-responsive behaviors by reversibly folding and unfolding due to water absorption at the film surface while the CSE with higher DS (CSE1.3 or CSE3.0) did not.

Moreover, Díaz-Payno and co-workers recently fabricated a smart bilayered 4D-printed multi-material system using alginate and hyaluronic acid hydrogels. The bilayered scaffolds was capable of self-bending upon differential swelling between the two zones, mimicking the curvature and multilayer cellular natural nature of the cartilage structure [38]. They also demonstrated that the degree of obtained curvature can be adjusted by tuning a number of parameters including the printing angle, the thickness of each layer, the CaCl_2_ crosslinking time, as well as the type of swelling solvent.

### 3.2. Temperature-Responsive Materials

**Figure 2:**
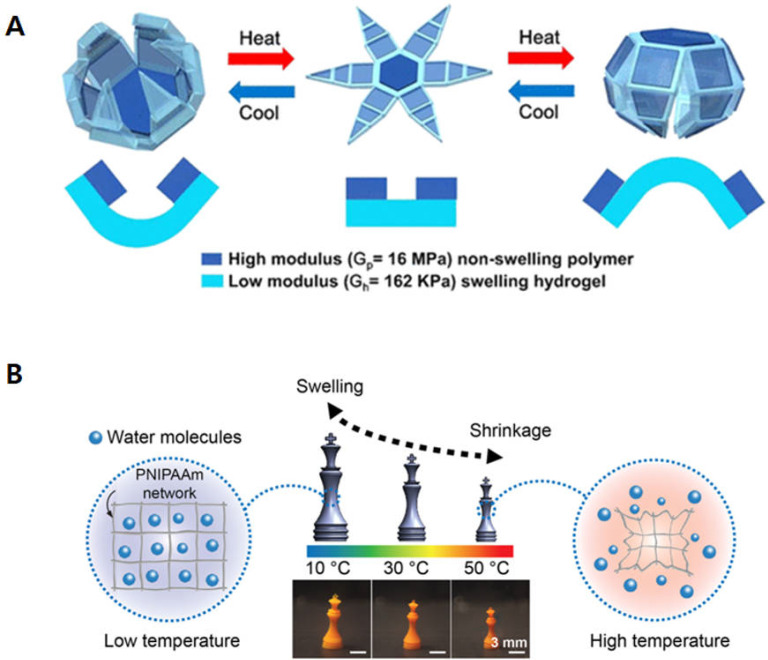
Schematics of the 4D-printed structure fabricated by temperature-responsive hydrogels. (**A**) The transformation behaviors of the thermo-responsive microgripper, reproduced with permission [42]. (**B**) Swelling behaviors of the 3D PNIPAAm hydrogel structures. When the temperature increased, the height of the structure decreased, reproduced from [43] under a creative common attribution 4.0 (https://creativecommons.org/licenses/by/4.0/, Accessed on 27 April 2024.).

Temperature is another common stimulus to transform bioprinted constructs. Temperature-responsive biomaterials are capable of transforming shape, size, or function in response to surrounding temperature variations [39]. Poly(*N*-isopropylacrylamide), or pNIPAM, is one of the widely used thermo-responsive materials. It has an unique property, which shows a reversible volume change in water at its low critical solution temperature (LCST, 32~35 °C) due to a coil-globule transition of the polymer network strands [40]. At a temperature below its LCST, the polymer becomes hydrophilic with an extended coil structure and cause it to swell, thus achieving shape transformation. Bakarich and colleagues fabricated a novel 4D-printed smart valve with thermo-responsive pNIPAM Ionic Covalent Entanglement (ICE) hydrogels as actuators. This innovative valve is engineered to regulate water flow by closing and opening when exposed to hot water (60 °C, contracted) and to cold water (20 °C, swelled), respectively [41]. Another study investigated the usefulness of photocrosslinked pNIPAM-co-acrylic acid (pNIPAM-AAc) to produce reversible self-folding soft photopatterned microstructures with temperature variation. These structures consisted of a rigid PPF layer atop a layer of pNIPAM-AAc. As the temperature surpassed 36 °C, the pNIPAM-AAc layer becomes hydrophobic, exposing the PPF segments inducing the opening and closing of microgrippers, orienting the PPF segments outward. Conversely, below 36 °C, the pNIPAM-AAc layer expands, causing the pNIPAM-AAc layer to face outward (Figure 2A) [42]. In addition, Han et. al. demonstrated the control of the swelling transition temperature of the pNIPAM structure by selective incorporation of an ionic monomer [43]. When the positively charged ionic monomer, methylamidopropyltrimethyl-ammonium chloride (MAPTAC), was incorporated into the polymer network, the transition temperature can increase up to 65 °C, depending on the concentration of the monomer. As a result, the 3D-printed hydrogel structure was swollen at 10 °C, whereas the structure gradually shrank when the temperature increased up to 50 °C (Figure 2B).

### 3.3. Electrical/Magnetic-Responsive Polymers

**Figure 3:**
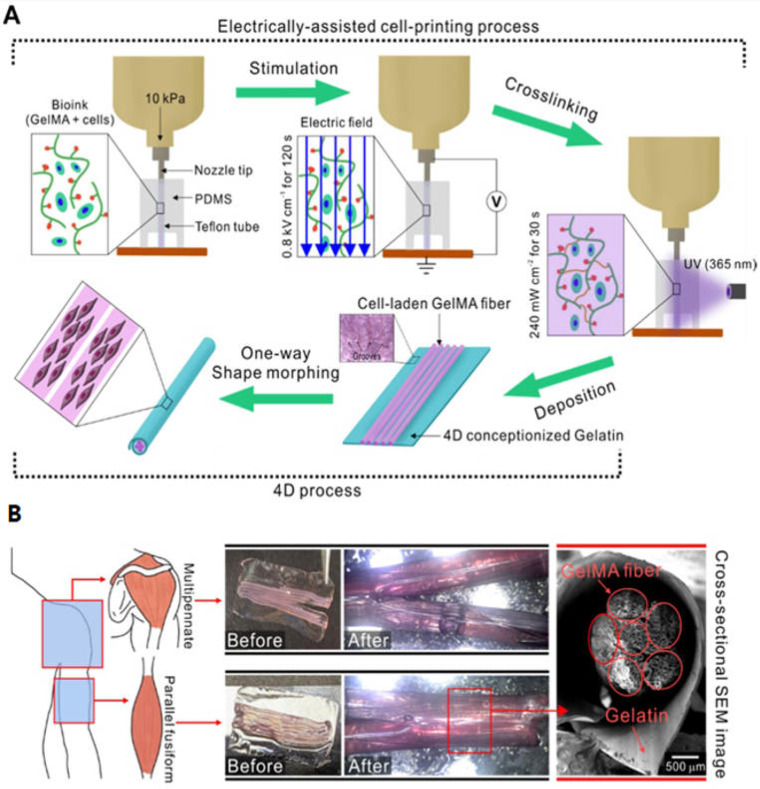
(**A**) Schematics of the fabrication of muscle fiber structures through the electrically assisted cell printing process. (**B**) SEM images of cell-laden GelMA fibers together with the shape morphing gelatin film, adapted from [45].

Electrically or magnetically responsive polymers contribute significantly to the realm of 4D printing by enabling structures to react to electrical or magnetic cues. These polymers undergo alterations in size, shape, or functionality when subjected to electrical or magnetic stimulation [7]. The most commonly used electro-responsive biomaterials are mainly conductive polymers such as polythiophene, polyaniline (PANI), and polypyrrole bioinks which can transform their shape in response to electric stimulus [44]. For instance, Yang et al. produced a cell-laden GelMA-based microfibril structure fabricated by electric-assisted 4D printing [45]. They developed an electric field-assisted 3D bioprinting process to fabricate cell-laden GelMA fibers. Upon electrical stimulation, cell alignment was induced, compared to the normally printed cell-laden structure (Figure 3A). The cell-laden GelMA fibers were deposited onto the gelatin film, which then formed a bundled structure consisting of microfibers (Figure 3B). Magnetic responsive hydrogels can offer a promising avenue for the controlled release of drugs and/or cells [46]. For instance, Mooney et al. engineered porous magnetic responsive hydrogels for drug delivery system or tissue engineering scaffolds. These active ferrogels exhibit volume change, exceeding 70% under moderate magnetic fields. This deformation and volume shift facilitates the outward displacement of water from internal pores, enabling the release of various drugs such as mitoxantrone, plasmid DNA, and chemokines from the scaffold [47].

## 4. Biomedical Applications for 4D Bioprinting

Four-dimensional printing is an extension of three-dimensional printing aimed at revolutionizing the biomedical and biotechnological industries by rectifying shortcomings of 3D printing. This technology offers a wide range of applications, from the creation of adaptive infrastructure and smart textiles to advancements in the medical field such as drug delivery systems and self-evolving biomedical devices. The potential of 4D printing is yet to be discovered fully.

### 4.1. Scaffold Preparation

This stimuli-responsive behavior of 4D structures can be used to cater to the scaffolding requirements of the human body. Structures that can change shape and function, including mechanical and electrical behavior in response to external stimuli, can be created using 4D printing technology. Research has been carried out on plant-based biopolymers that support cell attachment and are temperature-sensitive. Miao et al. created a shape-memory scaffold with SLA that supports human bone marrow mesenchymal stem cells (hMSCs) using soybean oil epoxidized acrylate. The scaffolds which were kept at −18 °C returned to their initial shape at 37 °C [22]. In a similar manner, PCL was used as a control and castor oil-based polymers were used to create porous complex scaffolds, which were then examined for hMSC adhesion and proliferation [23]. The time-dependent shape changes and cell modulation properties of polyurethane-based shape memory polymers with porous structures are being investigated. Wei Zhao et al. used magnetically stimulated shape memory composites in 4D printing to create a bio-designed tracheal scaffold. Shape memory polymer tracheal scaffold printed using a glass sponge structure demonstrated improved mechanical strength and performance [35]. Another group of polymers that are frequently used is lignin, cellulose, chitosan, hyaluronic acid, and alginate, etc. for the preparation of stimuli-based scaffolds [7,19].

### 4.2. Drug Delivery

The materials used in 4D printing exhibit stimuli-responsive behavior, which can be used for targeted and regulated drug delivery. Wang et al. examined the drug delivery characteristics of printed shape memory hydrogels (SMH) after loading the anticancer medication methotrexate into them. Because printed structures have a larger surface area than traditional ones, the in vitro studies demonstrated that drug release was higher in printed structures. Shape memory hydrogels (SMHs) were created by combining sodium alginate with Pluronic F127 diacrylate macromer (F127DA). It has been demonstrated that these SMHs have a greater rate of drug release and recovery ratio [48]. Melocchi et al. created devices for intravenous drug delivery using shape memory polymers based on polyvinyl alcohol that react to the presence of water. The device is injected into the body in a transient form, regains its shape in the presence of water, and is expelled through urine [49]. As previously noted, Breger et al. used a photolithographic technique to create thermoresponsive gripper structures from poly (*N*-isopropylacrylamide-co-acrylic acid) hinges. When applied to the body in a cold state, the grippers will close above 32 °C and adhere to the tissue, making it easier to administer therapeutic agents [42]. Even with these advances in using 4D printing to fabricate drug delivery systems, optimizing, sensing, and spontaneous drug release should be prioritized in order to improve treatment standards.

### 4.3. Sensors

Shape-memory polymers are being investigated by researchers as a potential treatment for hyperglycemia. The external conditions of temperature, humidity, electromagnetic waves, pH, and electrolyte intensity can serve as external stimuli for the composites [50]. In order to provide appropriate care and prevent infection, wounds must be monitored in real time. To monitor wounds in this regard, Mostafalu et al. developed smart wound dressing bandages with pH and temperature sensors [51]. Furthermore, Wang et al. presented a method for producing a reversible actuator that combines bilayer composites with structural design to self-sense strain. The conductive graphene polylactic acid (GPLA) facilitates the actuator’s reversible deformation ability, which is controlled by voltage. The deformation direction can be controlled by the wiggle structure [52]. Poly (ethylene glycol) diacrylate (PEG-DA) monomer was photopolymerized by Lv et al. to create a hydrogel film. The hydrogel films spontaneously deform and move in response to variations in humidity levels. It exhibits varying tones of pink color and fluorescence when illuminated at 365 nm under various relative humidity levels. Thus, it is possible to use these hydrogel films as humidity test strips [53]. Chen et al. reported on a high-performance integrated sensor-actuator that can execute simultaneous sensation and actuation. Gradient microgap structures enabled the decoupling of thermal stimulation and strain perception in nanocarbon/polylactic acid composites [54]. Chen et al. developed a novel multifunctional preceramic polymer composite with a shape memory effect for use in sensing and actuation applications. By modifying the material compositions, precursor inks are pyrolyzed into lightweight, self-shaping ceramics, creating complex structures [55,56]. Lee developed a UV exposure sensor to address the problem of skin cancer due to overexposure to UV radiation. The UV-responsive polymer is housed inside a flexible trilayer structure that is made up of two transparent protective layers. Additionally, as diabetes becomes a bigger issue, scientists are investigating glucose sensors [57]. Song et al. developed a chemo-resistive glucose sensor using platinum nanoparticles and the glucose oxidase enzyme. Glucoresponsive areas and low sheet resistance electrodes are produced by printing carbon nanotubes and polyaniline nanowires using an inkjet printer, respectively [55].

### 4.4. Medical Devices

Medical devices have the ability to identify, track, and predict illnesses and injuries. These substances, for instance, are utilized in intragastric balloons, percutaneous feeding tubes, and consumable electronic devices. However, the nondegradable nature of these polymers presents a challenge when using them. Zhang et al. developed a pH-responsive supramolecular elastomer composed of poly (acryloyl-6-aminocaproic acid) and poly (methacrylic acid-co-ethyl acrylate) as a means of preventing degradation. It is noncytotoxic and compatible with the intestinal environment, as demonstrated by cell experiments [58]. Soft robotics and 4D printing can work together to complete tasks with the least amount of human involvement. According to Zarek et al., shape memory polymers may be utilized in 4D printing to create customized medical devices like bifurcated stents that are made according to the anatomy of the patient. Because of the way these stents were made, they can change shape in response to variations in body temperature [59]. Based on a mathematical model, Waseem et al. created a T-shaped vascular bifurcation using 3D bioprinting and shape transformation capabilities. The tubular structures have a diameter in the millimeter range and assume the shape of a T junction when submerged in water. It showed remarkable cell viability and has the potential to act as a self-acting vascular graft [60].

### 4.5. Tissue Engineering

Three-dimensional bioprinting has made its way in meeting the expectations by regenerating various organs, scaffolds, and blood vessels, which can be used for replacing natural organs or body parts in the human body [61]. By addressing the scarcity of organs, 3D printing has revolutionized organ regeneration and raised the standard of care. A time-dependent dynamic process is added by 4D printing to the design fabrication process. To create near-infrared (NIR)-responsive 4D-printed nanomaterials, Cui et al. combined photothermal materials—black phosphorus quantum dots and polycaprolactone-polyethylene glycol copolymer—using two-photon polymerization [62]. For in situ tongue tumors, Su et al. created an NIR responsive tumoral injectable hydrogel using photothermal therapy (PTT). Because of the network formed by Ag_3_AuS_2_ nanoparticles (NPs), chitosan, and anionic proteins, the prepared soft materials exhibit a strong photothermal effect [63]. Hwangbo et al. used 3D printing in conjunction with one-way shape morphing to create a biomimetic collagen/hydroxyapatite scaffold, which is classified as 4D printing. The microchannel scaffold demonstrated significant promise for blood vessel growth and osteogenesis [64].

Using piezo-electric materials to mimic the electrical environment of a damaged bone is one of the main subcategories of bone regeneration. Four-dimensional-printed flexible bio-piezoelectric scaffolds have the potential to stimulate bone regeneration in a time-dependent manner, as per Chen et al. [65]. Wang et al. combined β-tricalcium phosphate/poly (lactic acid-co-trimethylene carbonate) (TCP/P(DLLA-TMC)) with black phosphorus nanosheets and osteogenic peptide to create photothermally responsive shape memory composite scaffolds. When NIR radiation is applied, the scaffold temperature rises to 45 °C and it changes shape to fit into irregular bone defects. After that, the temperature is lowered to 37 °C, simulating the characteristics of natural bone. In order to facilitate vascularization and skin flap regeneration, they employed the photothermal sensitivity of MXene nanosheets and the thermoresponsive behavior of pNIPAM hydrogels in the MXene-Hollow Fibers (MX-HF) scaffolds. The scaffolds facilitated angiogenesis and proliferation by demonstrating controlled delivery of vascular endothelial growth factor (VEGF) [66].

Cui et al. made a significant advancement in cardiac tissue engineering by using 4D printing to create a cardiac patch that can be used to treat myocardial infarction. When exposed to appropriate mechanical stimuli, the patch exhibits enhanced vascularization and self-morphing capabilities [67]. Neural tissue engineering experiments are also conducted with 4D-printed structures that feature microgrooves. A 4D-printed multi-responsive construct with stress-induced shape transformation properties was created by Miao et al. using stereolithography. After printing, the structures undergo solvent-induced relaxation and light-induced internal stress, which results in reversible shape-changing properties [68]. Kim et al. used the Digital Light Processing (DLP) technique to create trachea implants made of photocurable Silk fibroin hydrogel. After the implants were assimilated by the host, the implanted region showed signs of cartilage and epithelium growth [69]. Betsch and colleagues have also utilized 4D printing to aid in the creation of an artificial fibrocartilage. In this instance, the directional alignment of collagen fibers in agarose hydrogel has been achieved through the use of a magnetic mechanism. The hydrogels contain embedded streptavidin-coated iron nanoparticles that give the collagen fibers magnetic sensitivity. Because of their high compression moduli, these aligned collagen fibers are perfect for use in cartilage tissue engineering applications [70].

### 4.6. In-Vitro and In-Vivo Tissue and Organ Models

In vivo 4D modeling plays a crucial role in advancing our understanding of biomaterials. By incorporating dynamic flow—considering both spatial and temporal aspects—these models provide more accurate predictions of biomaterial behavior. Researchers can assess factors like mechanical stress, nutrient transport, and cellular responses. This informs material design, implant success, and tissue regeneration. Additionally, in vivo 4D models reduce the need for animal testing, offering a more humane and relevant approach to biomaterial research. In summary, in vivo 4D modeling enhances our understanding of biomaterials, facilitates predictive assessments, and promotes ethical research practices.

Organ transplantation models play a critical role in advancing transplant medicine. These models encompass both natural and synthetic scaffolds, as well as cultured organs. Scaffolds serve as structural frameworks that facilitate cell growth and tissue organization, ultimately forming functional organs. Recent progress includes the development of bioengineered organs using decellularized matrices, which retain extracellular matrix components while eliminating cellular antigens that might trigger immune responses. Additionally, cultured organs generated through tissue engineering techniques offer promising pathways for creating transplantable organs in vitro. Macchiarini et al. achieved successful transplantation of a tissue-engineered trachea into a patient, highlighting the clinical applicability of these innovative approaches [68]. The study by Yang et al. tried to make a skeletal muscle model using GelMA bioink through electric field assisted 3D/4D bioprinting [69]. Esworthy et al. also explained the advanced 4D printing technologies for brain tissue modelling [70]. Pingale et al. has carried out a detailed review on how 4D printing can enhance the applications of 3D printing by adding the time dimensions in simulating the physiological environments [71].

## 5. Current Challenges and Future Perspectives

Despite all these impressive achievements of smart biomaterials and 4D bioprinting technologies that are already established and currently under development, 4D-printed structures face obvious challenges in material design and successful fabrication technology.

One of the primary challenges in 4D printing includes the limitation of 4D printable stimuli-responsive materials. Moreover, the materials used for the 4D printing of biomimicking materials should also be biocompatible and biodegradable with suitable mechanical properties. Although metals, polymers, and ceramics, can be used as smart materials, only smart polymers are currently successful in 4D printing [71]. Furthermore, multiple stimuli-responsive materials are limited. While a number of multiple stimuli-responsive materials have been developed by using combinatorial stimuli such as temperature and pH, magnetic field and temperature, or pH and magnetic field [3], 4D-bioprinted materials that are capable of shape transformation in response to multiple physiological signals (e.g., neuronal regulation or humoral regulation), are preferred for biomedical application [72]. Therefore, developing novel multiple stimuli materials as well as making the existing responsive materials printable and biocompatible is another task for the future.

Embarking on the journey of 3D bioprinting and transitioning to 4D bioprinting presents significant challenges: It involves the optimization of materials, printing parameters, and the selection of cells and the need to optimize multiple variables (e.g., implant integration, coherent remodeling, vascularization) to produce native-like complex tissue structures [73]. Moreover, it is important to consider the relevance of the technology to clinical applications, including the needs of clinicians and patients. Achieving this requires translational research, which can be difficult in a new field where regulatory processes are also not well-established. The need to optimize the formulation and processing parameters of bioinks, in particular, ‘biocompatibility’ as well as ‘printability’ of bioinks, is a pre-requisite for an ideal bioink. To overcome these challenges, the emerging 4D bioprinting technologies including in situ bioprinting, may further advance bioprinting technology to eventual development of human-scale stimuli-responsive dynamic tissues and organs [74].

In addition, it is also important to consider and address ethical and regulatory aspects of this technology to ensure its responsible and safe use. In particular, the manufacturers of smart biomaterials for 4D printing have to follow corporate regulatory guidelines and compliance risks in planning a biocompatibility testing of devices for 4D-bioprinted structures. Inevitably, the determination of the biocompatibility of a device is a risk assessment exercise. Furthermore, the establishment of standardized protocols for both in vitro and in vivo biocompatibility testing of smart 4D-printed structures is a daunting task regarding their safety and efficacy in clinical settings. In this manner, advanced imaging technologies may be able to provide much more relevant information of the biocompatibility of biomaterials, especially with the help of advanced 3D imaging technologies such as the focus ion beam-scanning electron microscopy (FIB-SEM) [75]. As an alternative approach to in vitro 4D bioprinting, one may consider in vivo bioprinting (or in situ bioprinting) to avoid a transplantation requirement, which may reduce regulatory requirements for clinical translation [74,76].

**Figure 4:**
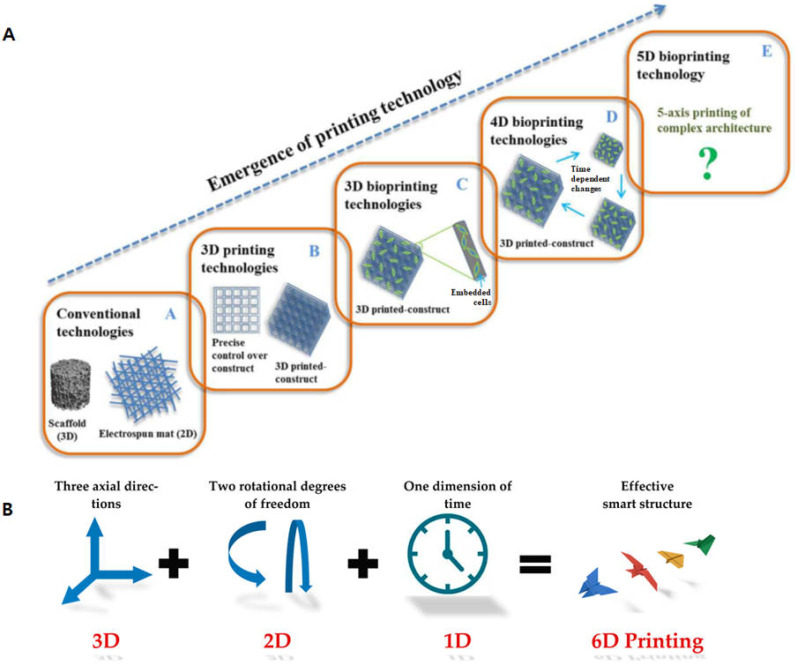
Evolution of bioprinting technology. (**A**) Multi-dimensional bioprinting and (**B**) emergence of 6D printing, reproduced from [78,79] under a creative common attribution 4.0 (https://creativecommons.org/licenses/by/4.0/, Accessed on 27 April 2024).

Nonetheless, overcoming these challenges is essential to unlock the vast potential of 4D bioprinting in various biomedical applications such as tissue engineering/regenerative medicine. Recently, 4D printing is now being evolved into 5D and 6D printing to further achieve complex morphology of the printed structures [77,78]. In 5D printing, the structure can be obtained by using three translational axes and two rotational axes, and 6D printed structures can be achieved with smart materials plus the additional dimension of time to obtain higher mechanical strength with less material [79] (Figure 4). Thus, these exiting printing techniques can be utilized to fabricate even more complex structures with advanced smart biomaterials.

The future of 4D bioprinting is likely to be marked by significant advances in a wide range of biomedical applications to revolutionize industries and contribute to more sustainable and breakthrough solutions to a variety of challenges. Obviously, the aforementioned innovative biomaterials may not see wide adoption to various biomedical applications in the near future in a standardized manner. Yet, it is clear that they will be able to provide a promising clinical outcome as researchers have made impressive progresses in smart biomaterials for 4D bioprinting in vitro as well as in vivo systems.

## Figures and Tables

**Table 1:** Comparison of different types of common 4D printing technologies [6,7].

Printing Type	Printing Speed	Cell Density	Cell Viability	Printing Resolution	Printing Cost	References
Extrusion based	Fast	High	Low (40~80%)	Low to moderate	Moderate	[2,8]
Inkjet printing	Moderate	Low (<10^6^/mL)	Moderate (>85%)	High	High	[9,10]
SLA	Fast	Moderate (<10^8^/mL)	Moderate (>85%)	High	Low to moderate	[11]
Laser assisted	Low to moderate	Moderate (<10^8^/mL)	High (>95%)	High	High	[12]

**Table 2:** Materials used in various stimuli-responsive applications.

Stimuli	Biomaterial	References	Behavior and Application	Limitations
Humidity-responsive materials	Poly (ethylene glycol) diacrylate (PEGDA)	[18]	These biological systems inspired the development of humidity-responsive materials that release or absorb moisture in response to changes in humidity. Systems composed of these materials are able to transform the sorption or desorption of moisture into driving forces for movement.	They tend to swell and degrade due to water absorption, impacting mechanical integrity. Stability under varying humidity conditions remains limited. Controlled drug release based on humidity changes is complex, and environmental sensitivity poses practical challenges.
	Cellulose	[19]		
	Polyurethane copolymers	[20]		
Temperature-responsive materials	Poly(caprolactone) dimethacrylate (PCLDMA)	[21]	These are the shape memory polymers and are widely utilized in drug delivery applications and tissue engineering applications, such as cell sheet engineering.	Temperature-responsive polymers have narrow temperature ranges, biocompatibility challenges, transition hysteresis, and limited stability.
	Soybean oil epoxidized acrylate	[22]		
	Polycaprolactone triol (Ptriol)	[23]		
	Poly(ether urethane) (PEU)	[24]		
	Poly(lactic acid)	[25]		
	Poly(*N*-isopropylacrylamide) (PNIPAM)	[26]		
	poly(*N*-vinylcaprolactam) (PNVC)	[27]		
	Collagen and ColMA, methylcellulose, agarose, Pluronic	[28]		
	Poly(ethylene glycol) based block polymers	[29]		
Electrical and magnetic-responsive materials	Iron(III)oxide (Fe_3_O_4_) nanoparticles containing mesoporous bioactive glass/poly(ε-caprolactone) (Fe_3_O_4_/MBG/PCL)	[30]	The potential of magneto-responsive materials in biomedical applications, has been demonstrated in many targeted drug delivery applications, where they offer minimally invasive, locally effective, and controlled therapeutic action	Common limitations for both include scalability issues, integration challenges, regulatory and safety concerns, and economic factors affecting market adoption.
	Magnetic nanocomposite scaffolds consisting of iron(III)oxide/PCL and iron(III)oxide/poly(ethylene glycol diacrylate) (PEGDA)	[31]		
	PCL/iron/doped hydroxyapatite (PCL/FeHA) nanocomposite scaffold	[31]		
Light-responsive materials	poly(*N*-isopropylacrylamide) (PNIPAM) functionalized with spirobenzopyran	[32]	It swells, shrinks or self-assembles upon photo-stimulation which is an area waiting to be explored. It has advantages, such as remote application with zero contact and ease of dose adjustment to control response strength.	They often exhibit low efficiency and limited sensitivity to specific wavelengths, reducing their versatility. Prolonged light exposure can degrade their performance, and they are sensitive to environmental changes like temperature and humidity. Slow response times and high production costs add to their drawbacks, along with complex fabrication and integration challenges.
	hydrogel system consisting of 4,4′-azodibenzoic acid (ADA), α-cyclodextrin, and dodecyl (C12)-modified poly(acrylic acid) (PAA)	[33]		
pH-responsive polymers	Poly(L-glutamic acid) (PGA), (PAA) (Dutta and Cohn, 2017), and)	[34]	pH-responsive polymer systems have been utilized in several biomedical applications, such as drug delivery, gene delivery, and glucose sensors due to their unique properties.	pH-responsive polymers have several limitations, including a narrow effective pH range and susceptibility to degradation in extreme pH environments. Their responsiveness can be affected by environmental factors like temperature and ionic strength, and they often have slow response times. The complexity and cost of synthesizing and processing these polymers, along with scalability issues, pose significant challenges. Biocompatibility concerns, such as toxicity and potential immune responses, limit their use in biomedical applications.
	Poly(histidine) (PHIS)	[23]		
	poly(acrylic acid)	[35]		
	poly(aspartic acid) (PASA)	[19]		

## Data Availability

Not available.
